# Ameliorating effect of *Alstonia scholaris* L. bark extract on histopathological changes following viper envenomation in animal models

**DOI:** 10.1016/j.toxrep.2018.10.004

**Published:** 2018-10-04

**Authors:** Rituparna Ghosh, Koushik Mana, Sumana Sarkhel

**Affiliations:** Department of Human Physiology with Community Health, Vidyasagar University, Paschim Medinipur-721102, West Bengal, India

**Keywords:** LD50, Lethal dose 50, AVS, Antivenom serum, AAS, Aqueous *Alstonia scholaris*, VRV, *Vipera russelli* venom, ALT, Alanine transaminase, AST, Aspartate transaminases, *Vipera russelli* venom, A*lstonia scholaris*, Nephrotoxicity, Hepatotoxicity

## Abstract

**Objectives:**

The primary symptoms associated with snake envenomation are both systemic and local. The local symptoms are characterized by pain, swelling, haemorrhage and myonecrosis at the site of bite. The present study investigates the ameliorating effect of the aqueous bark extract of A*lstonia scholaris* bark on viper venom induced histopathological and biochemical changes in liver and kidney of swiss albino mice models.

**Methods & materials:**

Swiss albino mice (20 ± 2)g were treated with sublethal doses(0.5 μg and 1 μg) of *Vipera russelli venom*(VRV) intraperitonially The following groups were assigned in the study-**Group I**(saline control); **Group II & III**(Venom treated-0.5 μg ie ¼ LD50 and 1 μg ie 1/2 LD50) and **Group IV &V**(Venom-0.5 μg and 1 μg respectively incubated with Aqueous *Alstonia scholaris* (AAS) extract; 200 mg/kg *bw*) and **Group VI** (Antivenom serum (AVS) (2 mg/ml) followed by 1 μg *Vipera russelli* venom (VRV). The animals were sacrificed and their organs were immersed in Bouin's fixative for 24 h and stained with haematoxylin/eosin and observed under the microscope. The serum samples were collected from the animals and tested for serum Alanine transaminase (ALT) and Aspartate transaminases (AST) following the method of Reitman & Frankel(1957) and serum creatinine.

**Results:**

The histological alterations observed in **Group II and III** liver sections were mainly pyknosis, karyorrhexis, cytoplasmic vacuolation, necrosis, fatty changes and hepatocytes atrophy. Sinusoidal dilatation, amyloidosis, portal vein thrombosis which was significantly reduced by AAS extract in **Groups IV and V**. A venom dose of 1 μg induced tubular cell acidophilia indicating cell damage, peritubular congestion, degenerating changes in the proximal tubules in the form of cytoplasmic vacuolations, partially destroyed bowman’s capillaries with dilated Bowman’s space in **Group III** that was significantly reduced by Aqueous *Alstonia scholaris* (AAS) (200 mg/kg *bw*) extract in **Groups IV** and **Group V**.AVS gave significant protection against venom induced action in **Group VI**.

**Conclusion:**

The present paper thus highlights the histopathological changes associated with *Vipera russelli* venom and systemic venom neutralization potential of Aqueous *Alstonia scholaris* (AAS) in animal models.

## Highlights

• In the present paper, the histopathological changes of liver and kidney tissues associated with *Vipera russelli* envenomation and systemic venom neutralization potential of aqueous *Alstonia scholaris* Linn bark extract in animal models has been discussed.
• Histological alterations observed in liver sections of venom treated groups were mainly pyknosis, karyorrhexis, cytoplasmic vacuolation, necrosis, fatty changes and hepatocytes atrophy.
• Histological changes in venom treated kidney tissues were cell damage, peritubular congestion, degenerating changes in the proximal tubules in the form of cytoplasmic vacuolations, partially destroyed bowman’s capillaries with dilated Bowman’s space after staining with haematoxylin and eosin.
• Both Antivenom and *Alstonia scholaris* Linn extract could significantly reduce the venom induced histological as well as biochemical changes (serum AST,ALT and creatinine) in the affected tissues.

## Graphical abstract

**Figure:**
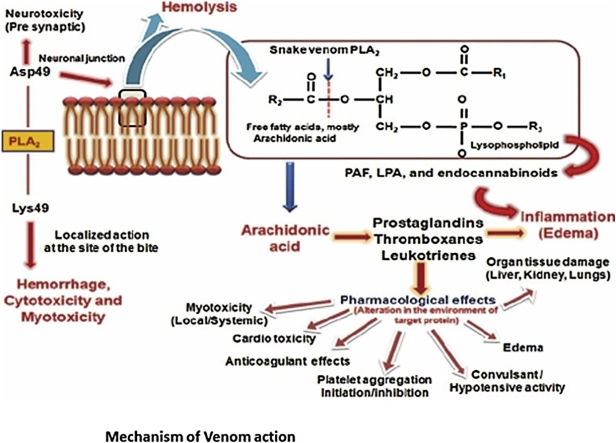

## 1 Introduction

Venomous and poisonous snakes are a significant cause of global morbidity and mortality. Nephrotoxicity and hepatotoxicity are the cardinal signs of snake envenomation. Viper venom is primarily vasculotoxic and has severe necrotising local effects. Persistent bleeding from fang puncture wounds, venepuncture or injection sites, and other new and partially healed wounds suggest that the blood is incoagulable. The primary symptoms associated with snake envenomation are both systemic and local. The local symptoms are characterized by pain, swelling, haemorrhage and myonecrosis at the site of bite. Hemostatic abnormalities are characteristic of viper bites and are the cause of the complications that lead to death. Accurate statistics of the incidence of snakebite and its morbidity and mortality throughout the world does not exist; however, it is certain to be higher than what is reported. Besides lethal effects snake envenomation also induces a wide range of local effects which can not be neutralized by anti serum. Nephropathy induced by snake venom was mentioned by Vikrant et al. [1]. Histo-pathological examination showed intense dose and time dependent abnormalities, including swelling glomerulus and tubular necrosis and damage as well as signs of intertubular medullary hemorrhage at early stages of envenomation in kidney sections [2]· Viper envenomation is associated with degeneration and necrosis. Focal glomerulonephritis is common. Viper venom causes nephrotoxicity and myonecrosis. Renal pathological changes include mesangiolysis, glomerulonephritis, vasculitis, tubular necrosis, interstitial nephritis and cortical necrosis.

The only specific treatment for snake envenomation is Snake venom antiserum (AVS) developed by Calmette (1894). Snake venom antiserum is of equine origin from the plasma of horse, mules etc that have been hyper-immunized against the most venomous snakes in India. It has been amply documented that intra and inter specific variation in venom composition can affect the neutralization capacity of antivenoms. Though a viable antidote snake venom antiserum does not provide enough protection against venom induced necrosis, haemorrhage and nephrotoxicity.

Research on plants as antisnake venom agents have several objectives. Plants are identified for their potency against venom and active principles are isolated and characterized.In traditional medicine there are several plants reported to have antivenom activity. Histopathological evaluation of animals treated with *Andrographis paniculata* extract following cobra envenomation showed mild muscular fiber regeneration, moderate edema, and absence of hemorrhage in the inoculated limb, in contrast to the group that received anti-venom serum alone. [3] Anti–snake venom (ASV) are immunoglobulins prepared by immunizing horses with the venom of poisonous snakes and subsequently extracting and purifying the horses' serum. They are the only effective antidote for snake venom. Antivenoms may be species specific (monovalent/monospecific) or may be effective against several species (polyvalent/polyspecific). Antibodies raised against the venom of one species may have cross-neutralizing activity against other venoms, usually that from closely related species.

*Alstonia* comprises about 40 species and has a pantropical distribution. There are about twelve species of the genus Alstonia. Alstonia boonei *De Wild* belongs to the family Apocynaceae. A wide array of chemical compounds has been isolated from *Alstonia boonei*. These include alkaloids, tannins, iridoids, and triterpenoids [4]. Chromatography of bark extracts of Alstonia boonei on silica gel plates with the solvent system AcOEt-MeOH-H_2_O (150 : 26 : 19) produced 6 separate spots with alkaloid reactions and the alkaloids isolated from the plant include echitamine and echitamidine, voacangine and akuammidine, Nα-formylechitamidine, and Nα-formyl-12-methoxyechitamidine [[5], [6], [7]].The present study addresses the neutralization potential of *Alstonia scholaris* bark extract on Viper venom induced histological and biochemical changes in liver and kidney sections.

## 2 Methods & materials

### 2.1 Venom

Lyophilized venom was commercially purchased from Calcutta Snake Park and preserved in dessicators at 4 °C in amber coloured glass vials. The snake venom was dissolved in distilled water and kept at 8 °C for 12 h and centrifuged at 3000 rpm for 30 min. The Supernatant was used as venom and kept at 8 °C for further use. The venom concentration was expressed as dry weight (mg/ml;w/v).

### 2.2 Antiserum

Lyophilized polyvalent snake venom antiserum I.P (batch no. 4066016) was commercially purchased from VINS Bioproducts Limited, Hyderabad, India. The concentration of antiserum was expressed in mg/ml.

### 2.3 Plant material

The dried bark of *Alstonia scholaris* Linn was commercially purchased from Debipada Das and Sons, Indigenous Crude Drugs Seller, Calcutta-700 007, India. The plant material was authenticated by taxonomist from the Department of Botany and Forestry, Vidyasagar University(Voucher specimen**(AS-SS101).** The plant material was grounded to powder and stored in thin plastic containers. The plant was extracted by cold maceration method.

### 2.4 Cold maceration method

Maceration was carried out in a closed conical flask for 72 h. 10 g powdered *Alstonia scholaris Linn* sample and 80% methanol as the extraction solvent were used. The suspension after maceration was centrifuged and the supernatant evaporated under reduced pressure. The solvent free methanol extracts were obtained. The concentration of the plant material was expressed as dry weight (w/v).

### 2.5 Percentage recovery yield of extraction

The percentage extraction yield (w/w) was calculated using the formula:

### 2.6 Percentage extraction yield for plant extract = [mass of extract (g)/mass of plant sample (g)] × 100

Aliquots of the extracts were stored in screwed cap vials at 4 °C–8 °C until further use. The extracts were re-dissolved in distilled water when required.

### 2.7 Animals

Swiss albino mice(20 ± 2)g was purchased commercially from authorised supplier of Vidyasagar University. The animals were kept in polypropelene cages, acclimatized and maintained in standard conditions(temperature 25 ± 2 °C; humidity 60 ± 5 °C and 12 h dark/light cycle). Food and water was provided *ad libitum.* All animal experiments were authenticated by the Department of Human Physiology; Vidyasagar University Ethical Clearance and in accordance with the Committee for the Purpose of Control and Supervision of Experiments(CPCSEA), Government of India **(clearance no. IAEC/Revised Proposal/SS01/2016 dt.01.05.2016).**

### 2.8 Acute toxicity studies

An acute toxicity test was performed according to the Organization of Economic Co-operation and Development (OECD) guideline 420 for testing of chemicals [8]. Swiss albino mice of both sexes, aged 6–8 weeks, old were used. A.scholaris aqueous bark extract was administered orally (only once) at a single dose of 2000 mg/kg at a rate of 20 ml/kg to both male and female mice (n = 12; 6 males and 6 females), whereas the control group received only 0.9% saline as a vehicle. After administration of A.scholaris aqueous bark extract, mice were observed for 24 h, with special attention given to the first 4 h and once daily further for a period of 14 days. The mice were weighed and visual observations for mortality, behavioural pattern (salivation, fur, lethargy, and sleep), changes in physical appearance, injury, pain, and signs of illness were conducted once daily during the period.

### 2.9 Viper venom neutralization studies

The following groups were assigned in the study-**Group I** (saline control); **Group II & III**(Venom treated-0.5 μg ie ¼ LD50 and 1 μg ie 1/2 LD50)) and **Group IV &V**(Venom-0.5 μg and 1 μg respectively incubated with Aqueous *Alstonia scholaris* (AAS) extract; 200 mg/kg *bw*) and **Group VI** (Antivenom serum (AVS) 2 mg/ml followed by1 μg *Vipera russelli* venom (VRV)).**Group I** received 0.1 ml of 0.9% saline intraperitonially. 0.1 ml of *Vipera russelli* venom (VRV) at two different concentrations were injected intra-peritonially in **Group II(0.5 μg)** and **Group III(1 μg).**

For neutralization of *Vipera russelli* venom (VRV) (0.5 μg & 1 μg) were preincubated with 200 mg/kg *bw* of *Alstonia scholaris* extract(AAS) in **Groups IV** and **Group V** respectively at 37 °C for 30 min followed by centrifugation at 3000 rpm for 10 min. **Group VI** received 2 mg/ml of Antivenom serum **(AVS)** 30 min prior to *Vipera russelli* venom **(VRV) (1 μg)** (Table 1).

**Table 1:** Venom treatment & neutralization with *Alstonia scholaris* bark aqueous extract(AAS).

Groups	Venom Dose(μg)	Plant extract(mg/ml) or saline(%) or AVS
Group I(n = 6)		0.9% saline
Group II(n = 6)	0.5 μg	
Group III(n = 6)	1 μg	
	Incubation at 37 °C for 30 min followed by centrifugation at 3000 rpm for 10 min	
Group IV(n = 6)	0.5 μg	200 mg/kg *bw*
Group V(n = 6)	1 μg	200 mg/kg *bw*
	AVS was given *i.p* 30 min prior to VRV	
Group VI(n = 6)	1 μg	2 mg/ml

**AVS:** Antivenom serum.

### 2.10 Histological studies

All the animals were sacrificed by cervical dislocation after 24 h. After abdominal dissection samples from the kidney were removed from each mice and washed in saline solution. These organs were immersed in Bouin's fixative for 24 h and washed. Sections were then dehydrated with graded alcohols(70%, 80%, 90% and Absolute alcohol). Following the routine procedure of the paraffin method, liver and kidney samples were processed up to paraffin blocks. Sections (5 μ thick) were made with WESWOX rotary microtome (Make:WESWOX OPTIK;Model MT1090 A 13023). Paraffin sections were deparaffized with xylene. De-paraffinized Sections (5 μ thick) were stained with hematoxylin and eosin for regular histological investigation. The preparations obtained were visualized using a light microscopy at a magnification of 400× [[9], [10], [11]].

### 2.11 Biochemical estimation of serum biomarker

For biochemical estimation, animals were divided into 6 groups(6 animals each). **Group I(**0.9% saline control) and **Groups II, III, IV, V and VI** received *Vipera russelli* venom(**Groups II & IV**-0.5 μg; **Groups-III, V and VI**-1 μg) intravenously. **Groups II(0.5 μg)** and **III(1 μg)** received only venom. **Group VI** received incubate of Viper venom VRV (**1 μg)** and antiserum**(2 mg/ml). Groups IV** and **V** received **venom** (**0.5 μg** & **1 μg**) and *Alstonia scholaris* extract(**200 mg/kg; *bw***) incubate. After 4 h blood was collected from swiss albino mice (from retro-orbital plexus) and allowed to clot for 30 min at 37 °C. The sample was centrifuged at 2000 rpm for 10 min. Colorimetric determination of serum alanine aminotransferase (ALT) and aspartate aminotransferase (AST) were estimated by according to the method of Reitman and Frankel(1957) [12]. Serum creatinine was estimated with the method of Toro *et al*(1975) [13] where the intensity of orange coloured complex formed by reacting alkaline picrate with creatinine was colorimetrically estimated.

### 2.12 Statistical analysis

Results were expressed as Mean ± SD. Results were analyzed using one way ANOVA. Differences were considered as statistically significant at P < 0.05 are compared to venom control. All data were analyzed using the Statistical Package for Social Sciences SPSS version 18.0(SPSS Inc, Chicago, USA).

## 3 Results

Acute oral toxicity studies with aqueous *Alstonia scholaris* extract at a dose of 2000 mg/kg had no adverse responses on the tested mice upto 14 days of observation. Physical observations showed no changes in the skin, fur, eyes mucous membrane, behaviour patterns, tremors, salivation, and diarrhea. There was no mortality observed at the tested dose nor was the weight loss in the rats affected. There was no significant changes in organ weight.

**Fig. 1:**
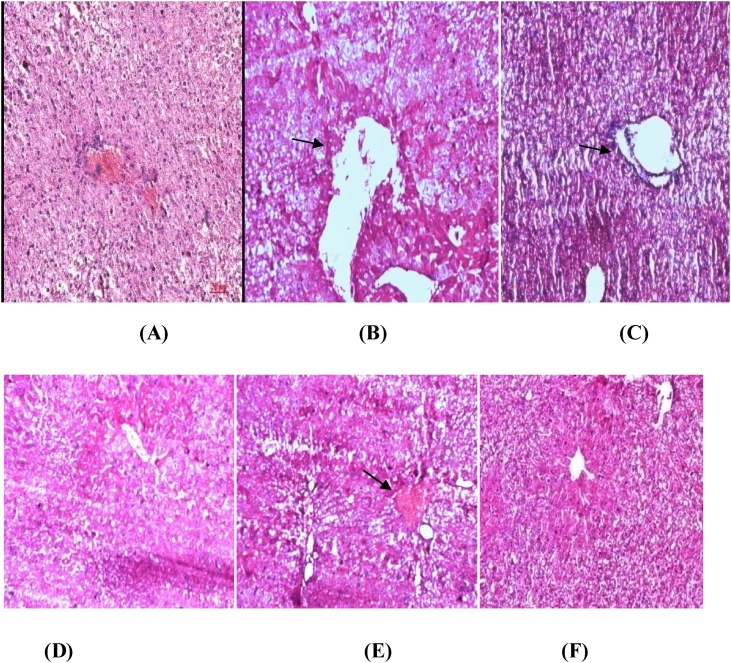
Liver sections of venom treated and Aqueous *Alstonia scholaris* (AAS) treated groups**:(A)**Group I-saline control**;(B&C)GroupII & III**-treated with 0.5 μg and 1 μg venom**;(D&E) Group IV & V**- treated with 0.5 μg and 1 μg venom + AAS(200 mg/kg bw**);(F)** Group VI -Treated with antivenom + VRV. Arrows indicate dialation of central vein,necrotic lesion and disintegrity of liver structure. Treatment with *Alstonia scholaris* aqueous bark extract have caused partial recovery.

The plant material recovered after cold maceration is 28%. Histological studies of the kidney sections of **Group II** and **Group III** showed degenerative changes in the kidney with disorganized glomeruli and necrotic tubules. Interstitial oedema and cellular infiltration were observed. There is diffuse and intense interstitial infiltration with mononuclear cells out of proportion to tubular degeneration. Peritubular congestion, degenerating changes in the proximal tubules in the form of cytoplasmic vacuolations, partially destroyed bowman’s capillaries with dilated Bowman’s space are evident in **Group II and III.** These effects have been reduced by *Alstonia scholaris* bark extract(200 mg/kg bw) in **Groups IV** and **Groups V** as compared to **Groups II** and **III.** Antivenom gave significant protection against venom induced changes in **Group VI(**Fig. 1**).**

**Fig. 2:**
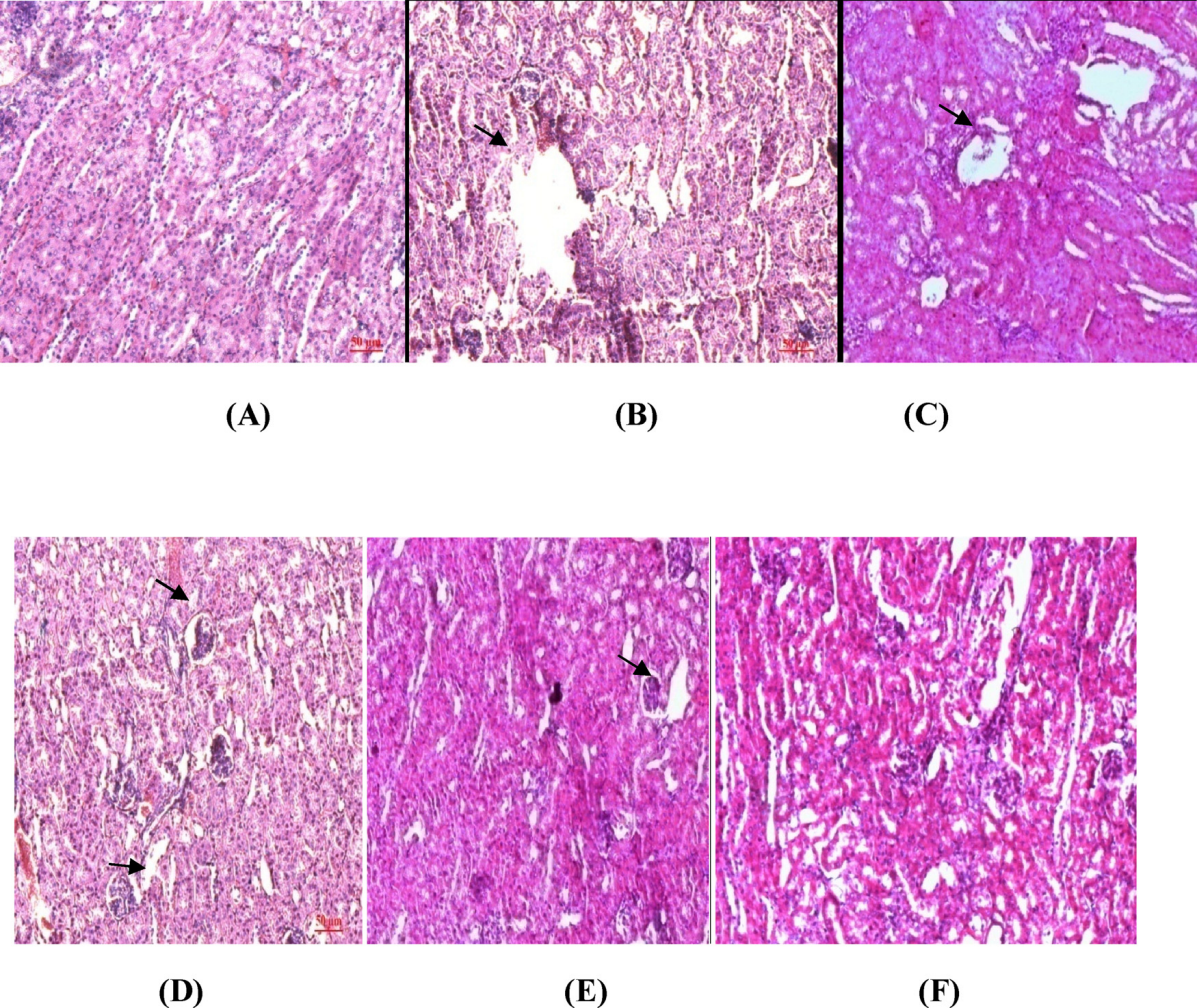
Kidney sections of the 6 groups **A)Group I**-saline control**;(B&C)GroupII & III**-treated with 0.5 μg and 1 μg venom**;(D&E) Group IV & V**- treated with 0.5 μg and 1 μg venom + AAS(200 mg/kg bw**);(F)** Group VI -Treated with antivenom + VRV.Arrows indicate congested glomerulus and increased capsular spaces. Treatment with Alsstonia scholaris aqueous bark extract have caused partial recovery.

The histological alterations observed in **Group II and III** liver sections were mainly pyknosis, karyorrhexis, cytoplasmic vacuolation, necrosis, fatty changes and hepatocytes atrophy. Severe necrosis with distended sinusoids and hepatic vacuolation are evident. Congestion of the central veins, congested liver sinusoids, leucocytes infiltration, cytoplasmic vacuolization and nuclear pyknosis, cellular swelling and necrosis of some cells are visible. Sinusoidal dilatation, amyloidosis, portal vein thrombosis which was significantly reduced by Aqueous *Alstonia scholaris* (AAS) extract in **Groups IV(**0.5 μg) **and V(1** μg). Antivenom gave significant protection against venom induced changes in **Group VI(Table-2)** (Fig. 2).

From Table 2 it is evident that Aqueous *Alstonia scholaris* (AAS) can significantly neutralize Viper venom induced rise in serum Alanine transaminase (ALT), Aspartate transaminase (AST) and creatinine in **groups IV and V.** Antivenom gave significant protection against venom induced changes in **Group VI** as compared to venom control.

**Table 2:** Neutralization of venom induced rise in serum Alanine transaminase (**ALT),** Aspartate transaminases (**AST) and creatinine with aqueous extract of** Aqueous *Alstonia scholaris* (**AAS)**.

Groups(n = 6)	Serum ALT(U/L)	Serum(AST(U/L)	Serum creatinine(mg/dl)
Group I (saline control)	12 ± 0.3	24 ± 0.02	0.53 ± 0.03
Group II(0.5 μg VRV)	57 ± 0.02	95 ± 0.05	1.9 ± 0.05
Group III(1 μg VRV)	75 ± 0.04	109 ± 0.03	2.2 ± 0.03
Group IV(0.5 μg VRV + AAS;200 mg/kg bw)	24 ± 0.02*	45 ± 0.01*	2 ± 0.02*
Group V(1 μg VRV + AAS;200 mg/kg bw)	35 ± 0.02*	50 ± 0.02*	1.8 ± 0.04*
Group VI(1 μg VRV + 2 mg/ml AVS)	19 ± 0.05*	22 ± 0.02*	0.62 ± 0.01*

* P < 0.05 as compared to venom control.

## 4 Discussion

Snakebite continue to be a major medical concern in India. Global health statistics for incidence of snakebite envenomations and their severity remain unknown or misunderstood. Morbidity and mortality resulting from snakebite envenomation also depends on the species of snake involved since the estimated fatal dose of venoms varies with species. Envenoming and deaths resulting from snakebite are a particularly important public health problem in the tropical world, with the highest burden in rural areas of South Asia, and sub-Saharan Africa. Snake bites, animal bites and stings are common occurrences, and snake bites are the highest among animal toxin poisoning. The high burden of snakebite and the fact that snakebite mostly occurs in rural areas, with less accessibility to professional health care and therefore rapid antivenom therapy, illustrate that adequate first aid treatments are of the utmost importance for achieving a positive outcome on both mortality and morbidity after a snakebite.

Snake bite is one of the most neglected public health issues in poor rural communities living in the tropics. Because of serious misreporting, the true worldwide burden of snake bite is not known. South Asia is the world's most heavily affected region, due to its high population density, widespread agricultural activities, numerous venomous snake species and lack of functional snake bite control programs. Despite increasing knowledge of snake venoms' composition and mode of action, good understanding of clinical features of envenoming and sufficient production of antivenom by Indian manufacturers, snake bite management remains unsatisfactory in this region. Immunotherapy is the only specific treatment for snake bite envenoming. Antivenoms are produced by fractionation of plasma obtained from immunized animals, usually horses. The success of antivenom therapy depends on the ability of immunoglobulins to bind, extract, and eliminate toxins present in the body.

Diverse mechanisms have been proposed to explain the kidney injury caused by viper venom, including rhabdomyolysis, hemolysis, shock, intravascular coagulation and a possible direct nephrotoxic effect [14]. More than 30 enzymes have been found in snake venom and twelve are found almost in all venoms in variable concentration. Viper venom posses thrombin like enzymes, fribinolytic enzymes and pro- coagulant. Phospholipase A_2_ is present in the venom of all families of poisonous snakes and is the enzyme that has been most widely studied. Snake venom (PLA_2_) enzymes induce a variety of pathological symptoms in animals even though have structural similarity and share common catalytic activity with non toxic mammalian pancreatic enzymes. On biniding specifically PLA_2_ can hydrolyses neighbouring phospholipids for inducing its pharmacological effects. Phospholipase A_2_ inhibits electron transfer at cytochrome C level and renders mitochondrial-bound enzymes soluble. It damages red blood cells, leukocytes, platelets, skeletal muscle, vascular endothelium, peripheral nerve endings, and the myoneural junction. Polypeptides, being smaller molecules, are rapidly absorbed into the systemic circulation and cause systemic toxicity in vessel-rich organs (e.g., heart, lung, kidneys, etc.) as well as at pre- and postsynaptic membranes. Excessive generation of ROS during arachidonic acid metabolism produces lipid peroxides leading to cellular injury. Vipers whose lethality is mainly attributed to the highly active enzymatic component, phospholipase A_2_ (PLA_2_) that hydrolyzes cellular phospholipids thereby releasing arachidonic acid. Oxidative metabolism of arachidonic acid results in the formation of potentially toxic reactive oxygen species (ROS) including superoxide and hydroxyl radicals. Acute renal failure complicates the course in 5% to 30% of victims of severe viper poisoning. Hyaluronidase helps spread of venom through tissues, and proteolytic enzymes are responsible for the local edema, blistering, and necrosis. No consensus exists on the single mechanism causing acute renal failure after viper bite. It is known, however, that viper venom induces several clinical abnormalities that favour the development of acute renal failure. These alterations include a varying degree of bleeding, hypotension, circulatory collapse, intravascular hemolysis, and disseminated intravascular coagulation with or without microangiopathy. Renal failure has been observed in patients a few hours after snakebite without hypotension, haemorrhage and rhabdomyolysis. Mesangiolysis [15], glomerulonephritis and vasculitis without immunologic clues indicate direct glomerular and vascular injury by the venom. The venom causes lysis of vascular smooth muscle, hydropic and vascular degeneration of proximal and distal tubular cells and cortical collecting duct with detachment from the basement membrane [16]. The pathogenesis of renal lesions in snakebite is complex involving both the direct action of venom on the kidney and the inflammatory effects due to the release of various endogenous cytokines and mediators. Phospholipase A, an important toxic component of the venom, stimulates hypothalamus-pituitary and immune axes to increase aderenocorticotrophic hormone, corticosteroid, arginine vasopressin and acute phase response [17].·Cortical necrosis has been documented in patients bitten by Russell’s viper [18]· In the present study congested glomerulus and increased capsular spaces and disintegrity of renal structure is evident from the kidney sections of **Group II** and **III** which are venom treated.*Alstonia scholaris* extract could also significantly neutralize viper venom induced rise in serum creatinine levels [19]. Increase in kidney parameters are similar as previous studies [20].

Liver is the primary detoxifying organ in the body could be affected by various types of toxic components in venom. Ghani et al, (2009) reported a large number of inflammatory cells, cellular necrosis, swollen and completely damaged hepatocytes of envenomated mice injected with ¼ LD50 and ½ LD50 *Naja n*i*gricollis* crude venom [21]. The venom of the Armenian adder (*Vipera raddei* Boettger) was tested for its ability to induce histo pathological changes in rabbits after long-term (once every 6 days for 30 days) intramuscular injection of the venom (0.35 mg/kg approx. 0.5 LD _50_), by light microscopic examination of some organs (liver, heart, kidney, adrenal, lung, spleen). *V. raddei* (Vr) venom induces changes including necrosis and edematous appearance with cellular infiltration and vacuolation. Liver sections showed vascular damage, significant quantities of hemosiderin and also presence of lympho histiocyte elements. In the present study *Alstonia* extract could significantly neutralize Viper venom induced rise in serum Alanine transaminase (ALT) and Aspartate transaminases (AST). Similar results were reported earlier with Echis pyramidum [22]. Transaminases and phosphatases are present in hepatocellular, which appears in circulation following the hepatocyte plasma membrane. Earlier *Vipera russelli* venom (VRV) induced pro-oxidant formation affecting the hepatocyte membrane resulting in increased transaminase levels have been reported [23]. Snake venom components, especially those of viper venoms, activate, inhibit or liberate enzymes by destroying cellular organelles [24]. Renal failure can be expected after envenomation by vipers with a serious course of intoxication. The underlying mechanisms are, besides glomerular hypofiltration by bleeding, decrease of intravascular volume by extravasation, microthrombi formation within consumption coagulopathy and vasoconstriction and direct nephrotoxicity of venom constituents: enzymatic destruction of renal marrow and renal tubules [25]. Earlier it has been reported that renal diseases caused by various snake venoms were characterized by raised urea, creatinine and potassium in oliguric patients. The disturbance in these values is used as an indicator of renal failure and impairment of the excretory function of the kidney which was ascribed to the nephrotoxic effect of venom [26]. In the present study dialation of central vein,necrotic lesion and disintegrity of liver structure are evident from histological sections which could be partially recovered by *Alstonia* extracts in **Groups IV** and **V**. The present study thus attempts to investigate the viper venom neutralization potential of aqueous extract of *Alstonia scholaris* bark probably by its effect on the biomarkers. Further studies are warranted regarding the bioactive components present in the extract and their role in neutralizing the viper venom activity. The probable mechanism of action of the plant has been investigated with the crude plant extract and so isolation and characterization of venom neutralizing components and their probable mode of action needs to be investigated and reported in the near future. The present study thus claims that there is a need for further investigation to identify active plant constituents against snake bite as an alternative remedy for existing anti serum. Structure elucidation of the active compounds enables synthetic production of the compounds on a large scale, thus providing cheap alternative toward snake venom antiserum.

## 5 Conclusion

Viper venom induces variable degrees of histopathological alterations in hepatic and renal which are main feature toxicity on tissue level and leading to death of envenomated people at higher doses. The present histopathological studies confirm that there is a marked reduction of injury in the liver and kidney sections after treatment with aqueous bark extract of *Alstonia scholaris* Linn. This reduction is marked in venom/extract group which may be attributed to the complexation of polyphenols with venom enzymes. Further studies on the mechanism of action of the plant material are warranted in future.

## Conflict of interest

Authors have no conflict of interest.

## Transparency document

Transparency document
